# The COVID-19 Treatment Landscape: A South African Perspective on a Race Against Time

**DOI:** 10.3389/fmed.2021.604087

**Published:** 2021-02-19

**Authors:** Candice Laverne Hendricks, Candice Herd, Marcel Nel, Gregory Tintinger, Michael Sean Pepper

**Affiliations:** ^1^Department of Medical Immunology, Institute for Cellular and Molecular Medicine, University of Pretoria, Pretoria, South Africa; ^2^Department of Internal Medicine, University of Pretoria, Pretoria, South Africa

**Keywords:** COVID-19, SARS-CoV-2, treatment, antivirals, antimalarials, high flow nasal oxygen, cytokine release syndrome

## Abstract

The pandemic caused by SARS-CoV-2 has infected more than 94 million people worldwide (as of 17 January 2020). Severe disease is believed to be secondary to the cytokine release syndrome (CRS or “cytokine storm”) which causes local tissue damage as well as multi-organ dysfunction and thrombotic complications. Due to the high mortality rates in patients receiving invasive ventilation, practice has changed from “early-intubation” for acute respiratory distress syndrome (ARDS) to a trial of non-invasive ventilation (NIV) or high flow nasal cannula (HFNC) oxygen. Reports indicating the benefit of NIV and HFNC have been encouraging and have led to more than 20,000 such devices being manufactured and ready for roll-out in South Africa (SA) as of July 2020. The need to identify drugs with clear clinical benefits has led to an array of clinical trials, most of which are repurposing drugs for COVID-19. The treatment landscape reflects the need to target both the virus and its effects such as the CRS and thrombotic complications. Conflicting results have the potential to confuse the implementation of coordinated treatment strategies and guidelines. The purpose of this review is to address pertinent areas in the current literature on the available medical treatment options for COVID-19. Remdesivir, tocilizumab, and dexamethasone are some of the treatment options that have shown the most promise, but further randomized trials are required to particularly address timing and dosages to confidently create standardized protocols. For the SA population, two healthcare sectors exist. In the private sector, patients with medical insurance may have greater access to a wider range of treatment options than those in the public sector. The latter serves >80% of the population, and resource constraints require the identification of drugs with the most cost-effective use for the greatest number of affected patients.

## Introduction

Coronavirus Disease 2019 (COVID-19) caused by SARS-CoV-2 emerged late in 2019 in China and has now spread to most countries across the world. The first case in SA was reported on 5 March 2020 and was followed by an initial strict lockdown from 27 March to 30 April 2020. Different levels of lockdown have been experienced since then and SA is currently experiencing its second wave as has been the case with many nations around the world. The Western Cape (WC), Gauteng, Kwa-Zulu Natal (KZN), and Eastern Cape have been at the center of the epidemic and the total number of patients who have tested positive to date in SA is 1,325,659 with 36,851 reported deaths (17 January 2021) (1). This translates into a case fatality rate of about 2.8% for SA which compares favorably with mortality rates reported in the USA and Italy of 3.5 and 16.7%, respectively (2). It has been proposed that the lower mortality rate may be due to the fact that SA has a younger population with a median age of 27.6 years, whilst in Italy this is 47.3 years (3).

It is well-recognized that the spectrum of disease caused by SARS-CoV-2 ranges from asymptomatic infection to severe acute respiratory distress syndrome (ARDS) and multi-organ failure. The latter is mostly secondary to systemic hyperinflammation, known as the cytokine release syndrome (CRS) or “cytokine storm” (4). The disease tends to be more severe in men, those individuals who are older than 60 years of age and those with associated comorbidities such as hypertension and diabetes mellitus. The high prevalence of human immunodeficiency virus (HIV) infection in SA is also cause for concern although the impact of HIV on outcomes in COVID-19-infected patients is currently unknown (5). In addition to sex, age and comorbidities, a number of prognostic factors associated with poor outcome have been identified since the start of the pandemic, and these include dyspnea, haemoptysis, heart and respiratory rate, and hematological or biochemical parameters including the neutrophil:lymphocyte ratio, serum LDH, albumin, lactate, NT-ProBNP, and bilirubin concentrations. These parameters have been incorporated into scoring systems such as the A-DROP (6) and COVID-GRAM scores (7) to predict potential outcomes in patients with COVID-19.

The COVID-19 pandemic has rightly focused attention on vaccine development with 10 vaccines rapidly entering clinical trials within a few months (8). A limited number of vaccines have received regulatory approval and vaccination has begun in several countries. By being a part of Covax, where resources are pooled for global distribution (8), SA is hopeful to start vaccinating frontline medical personnel in the next few months. Until the vaccine is widely administered both in SA and other countries, the disease will continue to be propagated and patients will require appropriate medical management. Strategies vary from one country to another as numerous agents are tested and either removed from or added to treatment protocols. Though many randomized control trials have been undertaken, much of current therapy is supportive or experimental in nature. The aim of this review is to provide information on the therapeutic options available to treat patients with COVID-19. The medications presented here have been grouped as followings: (i) therapy targeting viral attachment or replication; (ii) anti-inflammatory and immune-modulating therapy; (iii) supportive therapy; and (iv) novel cell-directed therapies. This is followed by two figures illustrating the point at which the various treatments might be effective as well as when to consider various forms of therapy during the progression of the disease.

## Therapy Targeting Viral Attachment or Replication

### Antimalarials

#### Hydroxychloroquine and Chloroquine Diphosphate

Chloroquine (CQ) and its analog hydroxychloroquine (HCQ) are antimalarial drugs with efficacy for certain chronic rheumatological diseases (9–12). Due to increasing resistance against CQ and a greater risk of toxicity when compared to HCQ (9), HCQ has now become the preferred treatment for rheumatological conditions, while CQ is used for malaria prophylaxis (11). The anti-viral effects of CQ have come to the fore in the last few decades (11) and *in vitro* activity of CQ against SARS-CoV-2 was reported by Wang et al. (13) The anti-viral effects are mediated through viral entry blockade (prevention of glycosylation of host receptors) (14), increasing the pH (alkalinization) of intracellular endosomes (which usually have an acidic environment) (13, 15) as well as immunomodulatory effects (14). A large observational study from New York city in the United States of America (USA) reported that the use of HCQ in 1,372 hospitalized patients did not increase or decrease the risk of intubation or death (16). The routine use of HCQ at this center was thus discontinued. Although one randomized trial from China has shown clinical benefit in a cohort of 62 patients, with a shorter period to resolution of pneumonia compared to the control group (80.6 vs. 54.8%) (17), another report of 30 patients again showed no difference between the treatment and control groups (18). A randomized trial of 150 patients attempted to determine whether initiation of HCQ for mild to moderate disease could decrease the negative conversion rate in patients (time from positive to negative test result) (19). Not only was no difference found in the HCQ group compared to controls, but a higher adverse event rate was shown with HCQ leading investigators to conclude that its use in mild-moderate disease should not be advocated. Borba et al. also showed that high doses of CQ, especially if used concomitantly with azithromycin and/or oseltamivir, resulted in a prolonged QTc interval on electrocardiogram (ECG) which may lead to fatal cardiac arrhythmias (9). A large multi-center retrospective observational study of 2,541 patients from Michigan in the USA showed improved survival in patients receiving HCQ, either alone or in combination with azithromycin (20). The authors claim that the early, standardized, safe dosing of HCQ as well as the use of an electrocardiogram (ECG) based algorithm to identify cardiac risk factors to guide the administration of HCQ, may have led to their positive results. A systematic review and meta-analysis of the use of HCQ with or without azithromycin (21) concluded that HCQ alone did not reduce mortality in hospitalized patients and that the addition of azithromycin greatly increased the mortality risk. The RECOVERY collaborative group then published their findings of HCO vs. standard of care in hospitalized COVID-19 patients and found no positive impact on mortality outcome with use of this drug (22). The World Health Organization (WHO) Solidarity trial (23) also found no benefit, and to date no data exists advocating for use of this drug in COVID-19 patients.

#### Artesunate

Artesunate is an artemisinin, a class of compounds originally derived from extracts of *Artemisia annua* (sweet wormwood) for the treatment of malaria (24), and has since been adopted by the World Health Organization (WHO). The use of artesunate has surpassed the use of chloroquines for the treatment of malaria and more recently for COVID-19 (25–27). Evidence for the use of *Artemisia* spp. extracts in traditional medicine dates back to 340 common era (CE) in Chinese culture (28) and is well-known across African cultures (29). Artesunate has been shown to have anti-viral properties against double- and single-stranded deoxyribose nucleic acid (DNA) and ribonucleic acid (RNA) viruses including human cytomegalovirus (HCMV), herpes viruses, hepatitis B and C, and related viruses (including Epstein-Barr virus) (30), providing the premise for deployment against SARS-CoV-2. The anti-viral mechanism of artesunate is thought to hinge on suppression of nuclear factor kappa beta (NF-κβ) activation (25). SARS-CoV-2 entry activates NF-αβ-regulated interleukin (IL)-6 amplifier, initiating the auto- and paracrine release of pro-inflammatory cytokines characteristic of the COVID-19 CRS (31). Artesunate could therefore mitigate the inflammatory response (32) and potentially improve patient outcome. Seven clinical trials have since been initiated to assess the efficacy of artesunate in different forms and administrations in reducing viral load and improving the prognosis of SARS-CoV-2-positive patients. A preliminary report documents a significant decrease in viral load and duration of hospitalization, and improved absorption of lung lesions in COVID-19 patients treated with 10 daily doses of 60 mg artesunate in addition to standard treatment (33).

### Antivirals

#### Remdesivir

Remdesivir (RDV) is a prodrug of a nucleotide analog, adenosine (13, 34, 35) and was initially studied in the context of the Ebola and other haemorrhagic viruses (15). The mechanism of action involves the inhibition of viral RNA-dependent RNA-polymerases (RdRp), terminating viral RNA synthesis which is required to produce new viral RNA and proteins (14, 36, 37). This mechanism confers wide anti-viral activity against many viruses including coronaviruses (SARS-CoV, MERS-CoV) and filoviruses (e.g., Ebola) (35–38). An initial report of 61 severely ill COVID-19 patients (from USA, Canada, Japan, Europe) in whom compassionate use of RDV was initiated, suggested improved outcomes and reduced mortality (34). Five randomized trials for RDV have been reported. The first was a large multi-center placebo-control trial from 10 hospitals in China that showed no statistically significant difference in clinical improvement between treatment and control groups (35). What it did show however was a numerical decrease in time to clinical improvement in patients treated with RDV, if treatment was commenced within 10 days of symptoms. The second trial from the USA included 1,063 patients and showed that patients on RDV had a shorter time to recovery and improved mortality rate compared to the placebo group (39). This led to its authorization by the European Medicines Agency (EMA), as well as in the USA and Japan for use in COVID-19 patients (38). The third trial compared a 5- to a 10-day course of RDV with no statistically significant difference being observed (40). The absence of a control group makes the findings difficult to interpret. The most recent trial from Spinner et al. included patients with moderate COVID-19 from 105 hospitals in the USA, Europe, and Asia (41). The trial showed benefit in clinical status after a course of 5 days of RDV compared to standard of care, but not in patients receiving 10 days of treatment. None of the trials were congruent in study design, inclusion criteria and outcome parameters. Based on these initial findings, a model comparing availability of ICU beds and projected numbers of infections was applied to SA to determine the effect of the addition of treatment with RDV to all patients admitted to ICU. By decreasing time spent in ICU, this model showed that thousands of lives could potentially be saved in SA by December 2020 (42). Unfortunately RDV has not been widely available in the public sector in SA despite the fact that the pharmaceutical company Cipla indicated that RDV would be available in SA from mid-2020 (43). The outcomes of the pivotal WHO_Solidarity trial however showed no effect in mortality outcomes in patients receiving the drug (23). There is insufficient evidence presently to confidently exclude RDV as a treatment option in COVID-19 patients. What is required from future randomized trials is to determine the correct dose and timing of the drug, and which routes of administration and combination therapies are most effective (44) bearing in mind that hepatic and renal adverse events have been reported (45, 46) and must be considered in risk-benefit decisions.

#### Lopinavir/Ritonavir

Lopinavir/ritonavir (LPV/r) is a well-known protease inhibitor used as part of the triple anti-retroviral (ARV) therapy for human immunodeficiency virus (HIV) (15). Protease enzymes are necessary for final cleavage of viral proteins prior to virion assembly (15). Data suggests that early initiation of this drug in patients with SARS improves mortality rates (47), and this effect was even greater when combined with ribavirin (48), another antiviral. In SARS-CoV-2, a randomized control trial using LPV/r in 199 patients with severe COVID-19 showed no benefit when compared to standard-of-care treatment (49). Some researchers felt however that the study was underpowered and that further trials are required before discrediting a widely available treatment (50). A systematic review and meta-analysis was performed by Alhumaid et al. and included 14 studies comparing LPV/r use in combination with or in comparison with other antivirals or standard of care (51). The authors concluded that LPV/r offered “no statistically significant advantage in efficacy.” The WHO Solidarity trial also showed no benefit (23). Further large randomized studies are awaited to adequately determine the effectiveness of this drug as well as the stage at which to implement it in the clinical course of COVID-19, especially in view of its side -effect profile (51, 52).

#### Interferon

Type I interferons, which include interferon-beta (IFN-β) and interferon-alpha (IFN-α), are antiviral cytokines with immunomodulatory properties. Interferon-beta (IFN-β) is a polypeptide drug that reduces neuronal inflammation and is approved for the treatment of relapsing-remitting multiple sclerosis (MS) while IFN-α has been used for the treatment of chronic hepatitis C (53–55). In the body, type I interferons are produced by most cells in response to viral infection and regulate antigen presentation, promote cytotoxic activity, and play a key role in the innate immune response (53, 56). IFN-β reduces the body's immune response by blocking the activity of type II interferons (IFN-gamma) and thus reducing inflammation (57). COVID-19 evades the innate immune system by suppressing endogenous IFN-β production, thereby increasing susceptibility to the development of severe symptoms. The use of cytokines with anti-viral activity such as IFN to treat patients infected with SARS-CoV-2 has been assessed in small clinical trials. Synairgen, a British Biotech Company, reported positive results from their phase 2 double blind placebo-controlled trials on 101 hospitalized COVID-19 patients with an inhaled form of IFN-β (SNG001) (58–60). Although tested in a relatively small number of patients, the pilot study results suggest that this agent reduces the risk of developing severe disease (such as requiring ventilation) by 79% and accelerated time to recovery. The study also showed reduced breathlessness as well as patients being twice as likely to recover from the infection by day 28 (60). The SNG001 study is currently being expanded to include 120 at home patients (SG016) and is also set to move on to phase III clinical trials (SG018) (Available from: https://www.synairgen.com/covid-19/). IFN-β1a also displayed anti-inflammatory effects *in vitro* for SARS-CoV-2 and MERS-CoV, and upregulated lung antiviral defenses in phase 2 clinical trials for asthma and chronic obstructive pulmonary disease (COPD) (58, 61, 62). A retrospective study of 456 COVID-19 positive patients who received a combination of lopinavir and ritonavir with or without INF-β1b demonstrated a reduced mortality rate and improved oxygen support when INF-β1b was included in the treatment regime (63). Further ongoing phase II clinical trials for INF-β1a include the open-labeled randomized INTERCOP clinical trial where 126 mild-moderate COVID-19 patients with signs of pneumonia will be included (55).

### Antibody Treatment

#### Convalescent Plasma

In patients who have recovered or are recovering from viral infections, neutralizing antibodies against the virus are found in the blood. Utilizing these antibodies in patients with active disease provides a potential means to contain the infection and in so-doing aims to decrease mortality (64, 65). Plasma is collected by apheresis and contains, in addition to neutralizing antibodies, certain anti-inflammatory cytokines and clotting factors, among many other proteins (66). These additional factors are thought to provide an added immunomodulatory mechanism against the COVID-19 CRS, but also in other viral infections (66), and also includes coagulation factors (67). Patients have to be ABO and RhD matched against the recipient and have to adhere to stringent criteria as with normal blood donation (68), as the risk of transfusion reactions is the same as with other blood products (65). Convalescent plasma transfusion (CPT) has been used with good effect in other infections such as severe acute respiratory syndrome (SARS-CoV), MERS-CoV, Ebola virus, and in the influenza A H1N1 pandemic, amongst others (67, 69).

The first trials using CPT for COVID-19 were reported from China and are summarized in a review by Rajendran et al. (64). The majority of patients were critically ill requiring intensive care, and infusion of CPT resulted in a measurable increase in the level of neutralizing antibodies. Although it appears that there may have been some benefit from the infusion, all patients were on additional therapies (e.g., antivirals and steroids) (70) making it difficult to determine whether the plasma contributed to the therapeutic effect (64). A further report showed that CPT did not impact on mortality when it was administered late in the course of disease, and this finding advocated for earlier administration in order to obtain clinical benefit (71). However, when given to patients with moderate COVID-19 in a randomized trial from India, no benefit was found in progression to severe disease, nor mortality (72). Initial reviews by Valk et al. (73) and Sarkar et al. (74), concluded that “low-certainty evidence” was available for a benefit from CPT in COVID-19 patients. Larger randomized controlled trials will be required to firmly establish the benefit of CPT and to establish guidelines on its most effective use, particularly its effect if administered very early in disease (75). Recent reviews have also failed to identify definitive evidence for clinical benefit (75, 76). Guidelines have been prepared by the International Society for Blood Transfusion (ISBT) providing guidance for the preparation and use of CPT in COVID-19 patients (77), with a special report focusing on the implementation of these guidelines in low and middle income countries (LMIC) (68). The South African National Blood Service (SANBS) has initiated collection of CPT for the purposes of a national clinical trial for COVID-19 patients (78).

#### Monoclonal Antibody Specific to SARS-CoV-2

Monoclonal antibodies (mAb) are produced as part of the body's response to pathogens and are specific to a single epitope of the pathogen. These Abs maintain memory B-cells which will mount an immune response following subsequent encounters with the same pathogen (79). SARS-CoV-2-infected individuals generate these unique sets of mAbs against the virus, which can be isolated and evaluated for efficacy before selection and mass-production for therapeutic purposes (80). The efficacy of mAbs in convalescent plasma differs between donors; following selection, mAb therapy could be prepared as an off-the-shelf product from the most potent mAb combinations against SARS-CoV-2. Preliminary studies have yielded promising results in the search for mAb or mAb cocktails targeting the SARS-CoV-2 S-protein (81–85). These studies may also provide insight into vaccine design to determine which mAb will illicit the most potent SARS-CoV-2 neutralization in uninfected individuals (81). The first randomized, placebo-controlled, double-blind Phase I clinical trial using a mAb (Ly-CoV555; NCT04411628) began treating patients in June 2020 (86). A multitude of studies have since emerged in the clinical trial space, leading to the development of a COVID-19 antibody therapeutics tracking tool by the Chinese Antibody Society (87) According to the tracker, 107 SARS-CoV-2 mAb treatments are in varying stages of development, of which 23 are clinical trials in Phase I, II, or III (https://chineseantibody.org/covid-19-track/; accessed 12/01/2021). Initial results using the Ly-CoV555 mAb (NCT04427501) indicated that treatment of COVID-19 outpatients may accelerate viral load clearance (88). However, more recent results (NCT04501978) showed that Ly-CoV555 when administered with RDV demonstrated little efficacy (89) which may result from insufficient participants for robust statistical analysis between groups, according to the authors. The mAb cocktail REGN-CoV2 produced by Regeneron Pharmaceuticals Inc. was assessed in COVID-19 patients and demonstrated effective reduction in viral load when administered early in infected patients (NCT04425629) (90) after having been assessed in rhesus macaques and hamsters in which it demonstrated marked viral load reduction (91).

## Anti-Inflammatory and Immune-Modulating Therapy

### Steroids

Corticosteroids are known to suppress inflammation (92) and provide blood pressure support, and were used in the SARS and MERS-CoV epidemics. Upon review of outcomes however, Russel et al. reported increased mortality, delayed clearance of infection, psychosis, and diabetes as some of the complications (14, 93). The authors argued that hypotension in respiratory related shock is more likely to be associated with increased intrathoracic pressure from invasive ventilatory support, and steroids were thus not thought to provide benefit. These arguments were countered by Shang et al. who claimed that benefit had been seen in previous cohorts of patients with viral pneumonias, particularly in severe disease (94). Based on these findings, an expert consensus document was released guiding the use of these drugs until evidence from a randomized control trial could be published. On the 16th June 2020, a breakthrough study was reported. Based in the United Kingdom (UK), the RECOVERY trial showed clear benefit from the use of dexamethasone in patients with COVID-19 requiring oxygen therapy (95, 96). There was a decrease in mortality by one third in ventilated patients, and by 20% in those on oxygen therapy. The average age of the patient cohort was 59 years; upon analysis of older age groups, the beneficial effect was less clear (97). The exact dose as well as the timing and duration of delivery are areas that were not addressed in the trial. This is particularly important, as dexamethasone has effects on B-cell antibody production as well as T-cell functioning and its use must be controlled in order to obtain the appropriate benefit (92). However, as dexamethasone is a relatively inexpensive and widely available drug, this was a very important finding and is encouraging for patients with severe COVID-19 disease.

### Colchicine

Colchicine, an inexpensive anti-inflammatory agent used for the treatment of gout, has been used in small clinical trials as adjunctive therapy for patients with COVID-19 (98, 99). Colchicine works by interfering with COVID-19 related manifestations such as hyper-inflammation, and inhibits inflammasome activation, neutrophil chemotaxis and the production of pro-inflammatory cytokines such as interleukin-1beta (IL-1β) and interleukin-18 (IL-18) (98, 100, 101). In a small study of 5 patients who received colchicine for iatrogenic allogenosis (IA) or foreign modeling agent reactions (FMAR), COVID-19 patients displayed mild symptoms and did not require hospitalization (98). Another study in Italy compared 262 COVID-19 positive patients who either received standard of care (SoC) treatment or colchicine; a significantly higher survival rate was observed in patients who received colchicine (102). Four ongoing clinical trials include the Colchicine Coronavirus SARS-CoV2 trial (COLCORONA), The Greek Study in the Effects of Colchicine in COVID-19 Complications Prevention trial (GRECCO-19), the Colchicine Counteracting Inflammation in COVID-19 Pneumonia trial (COLCOVID-19), and the Effects of Colchicine on Moderate/High-risk Hospitalized COVID-19 Patients trial (ECLA PHRI COLOVID) (99, 101, 103–106). More recently, colchicine treatment has also been added to the RECOVERY trial in the UK where at least 2,500 patients will be recruited (Available from: https://www.recoverytrial.net/news/colchicine-to-be-investigated-as-a-possible-treatment-for-covid-19-in-the-recovery-trial) The GRECCO-19 clinical trial has thus far reported a statistically significantly prolonged time to clinical deterioration as well as a decrease in the number of patients requiring intubation and ventilation when compared to the placebo group at 21 days (107). A randomized double-blinded study of 38 hospitalized patients showed a decreased need for oxygen supplements, hospitalization and reduced C-reactive protein (CRP) when treated with colchicine (108, 109). Although data on the use of colchicine on Covid-19 outpatients are limited, with some authors initially advising against the use of colchicine to treat severe coronavirus infections (110), these results provide support for further investigation of colchicine therapy to reduce hospitalization as well as adverse outcomes (109).

### Treatment for Cytokine Release Syndrome

#### Tocilizumab

SARS-CoV-2 causes a CRS with interleukin-6 (IL-6) being implicated as one of the major cytokines (111, 112). IL-6 is a “multi-effective cytokine” (111), with pro- and anti-inflammatory properties, and is critical for normal host immunity. Tocilizumab is a recombinant human antibody which binds to the IL-6 receptor (113), and has been widely used in the management of CRS in other settings (15). It is approved for CRS secondary to chimeric antigen receptor T-cell (CAR-T) therapy, but is also used for rheumatic diseases (4, 113). Efficacy was initially shown in small cohorts of patients with COVID-19 (114, 115). Toniati et al. evaluated 100 patients prospectively post administration of intravenous (IV) Tocilizumab (116). Of those in intensive care, 77% improved, while 66% of patients treated in the general ward showed improvement in respiratory function. These findings were supported by subsequent reports (117, 118). One randomized trial from Italy however showed no benefit in progression of disease compared to standard care (119), whilst another from the USA showed an advantage in progression to mechanical ventilation but not overall mortality (120). The most recent systematic review and metanalysis showed a decreased mortality rate when Tocilizumab was used, but no effect was seen in COVID-19 severity and length of hospital stay (121). In low and middle income countries (LMIC), the cost of this drug may be prohibitive and even when available, patients in whom the most benefit can be derived would have to be carefully selected. Presently, the optimal timing for introduction is not known and side effects of the drug such as liver dysfunction must be considered when assessing patient eligibility (122).

#### Anakinra

Anakinra is an interleukin-1 (IL-1) receptor agonist approved for use in treating rheumatoid arthritis. The IL-1 family of pro-inflammatory cytokines are important coordinators of the innate immune response and are integral to the COVID-19 CRS (123). The pro-inflammatory effects of IL-1 are mitigated by the binding of IL-1 receptor agonists such as anakinra to the IL-1 receptor (123). Preliminary results indicate control of inflammation in a few severe COVID-19 cases (124–128), notably reducing mortality and the need for mechanical ventilation (129–133). As of 12 January 2021, 28 clinical trials registered on clinicaltrials.gov were recruiting patients for the use of anakinra to treat severe to critical COVID-19 patients.

### Managing Excessive Angiotensin II

The binding of SARS-CoV-2 to ACE2 and the subsequent endocytosis of the virus-receptor complex decreases the availability of ACE2 for binding to angiotensin II (134, 135). The resultant increased levels of angiotensin II have been shown to mediate lung injury/inflammation, fibrosis, and myocardial dysfunction (134, 135). Under normal circumstances, angiotensin II would be converted to angiotensin (1-7) upon ACE2 binding, with resultant vasodilatation. Other effects of angiotensin (1-7) are antifibrotic, anti-proliferative, and diuretic (136). Hypertensive patients on ACE inhibitors and angiotensin receptor blockers (ARB) were initially thought to be at higher risk of developing COVID-19 as these drugs increase expression of ACE2. Studies done in Italy (137) and New York (138) have however found no independent risk related to the presence of these drugs and the risk of contracting COVID-19. On the contrary, a different school of thought now believes that treatment with ACE inhibitors and other drugs which block the renin-angiotensin-aldosterone system (RAAS) can in fact be beneficial (136, 139). The premise is that pro-inflammatory cytokine release and levels of angiotensin II can be reduced by decreasing production of angiotensin II through ACE inhibitors or by blocking the binding of angiotensin II to the ATR1 receptor through ARB blockers and therefore increasing binding to ACE2 with production of angiotensin 1-7) and may mitigate the harmful effects of these molecules. In particular, Losartan, by blocking the AT1R receptor through which angiotensin II mediates its vasoconstrictor effects, is a potential COVID-19 treatment option (140). This has also been the basis for research into the use of recombinant ACE2, which has been shown to impact myocardial remodeling and injury response in animal models (134). Providing recombinant human ACE2 (rhACE2) (141) may allow SARS-CoV-2 to bind to it instead of to membrane bound receptors. This would allow conversion of angiotensin II to angiotensin 1-7, and in so-doing mitigate the risk of developing ARDS (139, 141, 142). Phase I and II clinical trials with a rhACE2 for ARDS (not secondary to SARS-CoV-2) have shown decreased Ang II and IL-6 levels with a resultant increase in Angiotensin (1-7) and (1-5) (141). The limited number of subjects however made deductions on clinical benefit difficult. After initial safety studies (143), the first infusion of rhACE2 for SARS-CoV-2 was in a 45 year old woman from Austria with severe disease (142). The patient had viral clearance initially from the serum, followed by the nasal cavity and lung. Neutralizing antibody levels were not affected and the patient recovered. The authors concluded that systemic spread of the virus could potentially be blocked by this drug. These viral load reductions were also found in a second infused patient (143).

## Supportive Therapy

### Oxygen

Patients who require admission to hospital are usually those with severe COVID-19-related disease, defined as clinical evidence of pneumonia with either a respiratory rate > 30/min, signs of respiratory distress or peripheral oxygen saturation < 90% on room air. These patients often present with respiratory failure and hypoxemia or develop worsening respiratory failure while in hospital. The mainstay of treatment for these patients is supplemental oxygen that may be administered via oxygen cannulae, face masks or by means of high flow nasal oxygen (HFNO). The use of HFNO has become an important component of supportive therapy for patients with COVID-19 pneumonia (144, 145). Based on this more than 20,000 such devices were manufactured and ready for roll-out in South Africa (SA) as of July 2020 (146).

HFNO allows adequate oxygenation to be maintained and may decrease the requirements for non-invasive ventilation and endotracheal intubation. This has recently been confirmed to be true in the SA setting by Mendelson et al. who described their experience in Cape Town (147). The authors did however caution that the HFNO initiation should be governed by strict criteria, including a constant and stable oxygen supply. The clinical response to HFNO can be monitored using the ROX index (Oxygen saturation/F_i_O_2_ (%) x respiratory rate). A ROX index > 4.88 at 2, 6, or 12 h predicts successful treatment with HFNO (148). In addition to HFNO, the proning of awake patients or “conscious proning” has been shown to improve oxygenation (147).

### Management of Thrombotic Complications

The association between inflammation and coagulation is well-established and is linked to the presence of pathogen-induced cytokines that activate procoagulant pathways (149). In seriously ill patients, factors such as immobilization, underlying health problems and indwelling catheters amongst others, further exacerbate this clinical state (150). Sepsis-induced coagulopathy (SIC) is a set of diagnostic criteria used by The International Society of Thrombosis and Haemostasis (ISTH) to identify patients at risk of developing disseminated intravascular coagulopathy (DIC) (149). In DIC, widespread coagulation in large and small vessels leads to thrombotic complications with ischemia, while consumption of clotting factors leads to an increased risk of bleeding. Coagulopathy has been shown to be associated with a worse outcome in patients with COVID-19 (151, 152), with a thrombotic rather than a bleeding phenotype (152). The pathophysiology is thought to be multifactorial with endotheliopathy, antiphospholipid syndrome, auto-immune mechanisms, microvascular thrombosis, and complement activation being some of the proposed mechanisms (150, 153). The binding of SARS-CoV-2 to the ACE2 receptor on endothelial cells is a unique feature leading to a microangiopathy (149). The increased risk of venous thromboembolism associated with COVID-19 prompted many physicians to start using prophylactic and even therapeutic doses of anticoagulants such as heparin and low molecular weight heparin (LMWH). This may also be guided by the measurement of circulating D-dimers which, if significantly elevated, indicate the need for therapeutic anticoagulation. The ISTH and the American Society of Hematology (ASH) therefore recommend the initiation of thromboprophylaxis in COVID-19 patients requiring hospitalization with an escalation to therapeutic doses if clinically indicated (150). Subsequent studies have shown that the use of low molecular weight heparin (LMWH) improves survival in patients with severe disease (154, 155). A recently published observational cohort study from Italy also showed that increasing doses in severely ill patients in intensive care, rather than giving prophylactic doses, had a significant survival benefit (156). Only 3% of patients had major bleeding episodes and these were not fatal.

## Novel Cell-Derived Therapies

Cell (and cell-derived) therapies for the treatment of SARS-CoV-2/COVID-19 are still in the realm of experimental treatments or clinical trials, with mesenchymal stromal/stem cell (MSC) trials being the most prolific (157, 158) (108 trials across platforms, with few results posted to date). Infusions of immunomodulatory MSCs in preliminary studies successfully reduced the CRS and reversed the symptoms of COVID-19 with no side effects (159–161). The immunomodulatory properties of MSCs are proposed to originate from their secretion of pro- and anti-inflammatory cytokines, interaction with immune cells, and ability to potentially replace damaged cells by differentiation (157, 158, 162–164). CAP-1002 cells (cardiac progenitor cells in culture) have been administered experimentally to COVID-19 patients with acute respiratory distress syndrome (ARDS), although all patients also received Tocilizumab prior to CAP-1002 infusion (165). These experimental developments have paved the way for regenerative medicine/cell therapy approaches to potentially treat not only COVID-19 symptoms, but also the fibrotic aftermath of SARS-CoV-2 infection, as reviewed extensively by Basiri et al. (166). In addition to MSCs, MSC exosomes and conditioned medium (containing secreted immunomodulatory factors), fibroblasts, umbilical cord blood stem cells, and platelet-rich plasma treatments make up the bulk of the clinical trial landscape for cell therapies being assessed to treat and reverse moderate to critical COVID-19 symptoms. Cell therapies to reduce viral load are also in clinical trials, and proposed therapies range from SARS-CoV-2-specific T-cells (167) and T-cell exosomes to natural killer (NK) cell therapy products. T-cell exosomes have recently gained traction for their apparent capacity to effect T-cell responses (168, 169) and are being tested for the ability to overcome COVID-19-associated immune dysregulation. T-cells and NK cells are instrumental in clearing virus-infected host cells and provide immunological memory for subsequent encounters with the virus (167, 170), which is important for long-term immunity. Such therapies aim to ameliorate cytotoxic T-cell and NK cell exhaustion observed in COVID-19 patients (171, 172).

### Figures

**Figure 1:**
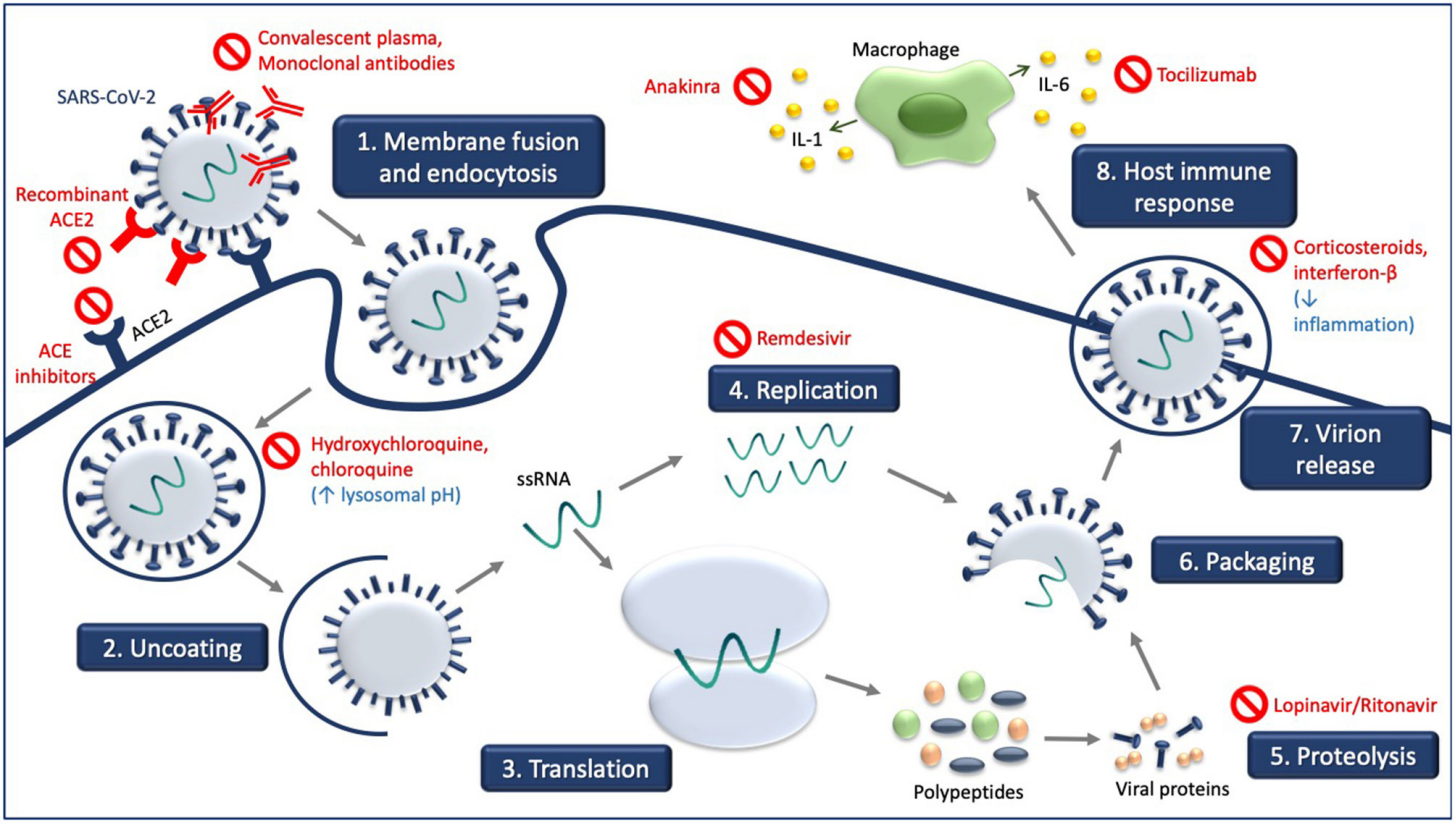
Experimental treatment strategies targeting different steps of SARS-CoV-2 life cycle and consequences of SARS-CoV-2 infection. Receptor-mediated entry of SARS-CoV-2 is blocked by recombinant ACE2 and ACE inhibitors which compete with SARS-CoV-2. Hydroxychloroquine and chloroquine increase in lysosomal pH. Viral genome replication is blocked by remdesivir, and the processing of SARS-CoV-2 polypeptides into viral proteins by proteolysis is blocked by lopinavir/ritonavir. Convalescent plasma and monoclonal antibodies target the released virus and facilitate clearance of the SARS-CoV-2 virus. Treatment with corticosteroids and interferon-B decrease inflammation associated with COVID-19, as do anakinra and tocilizumab.

**Figure 2:**
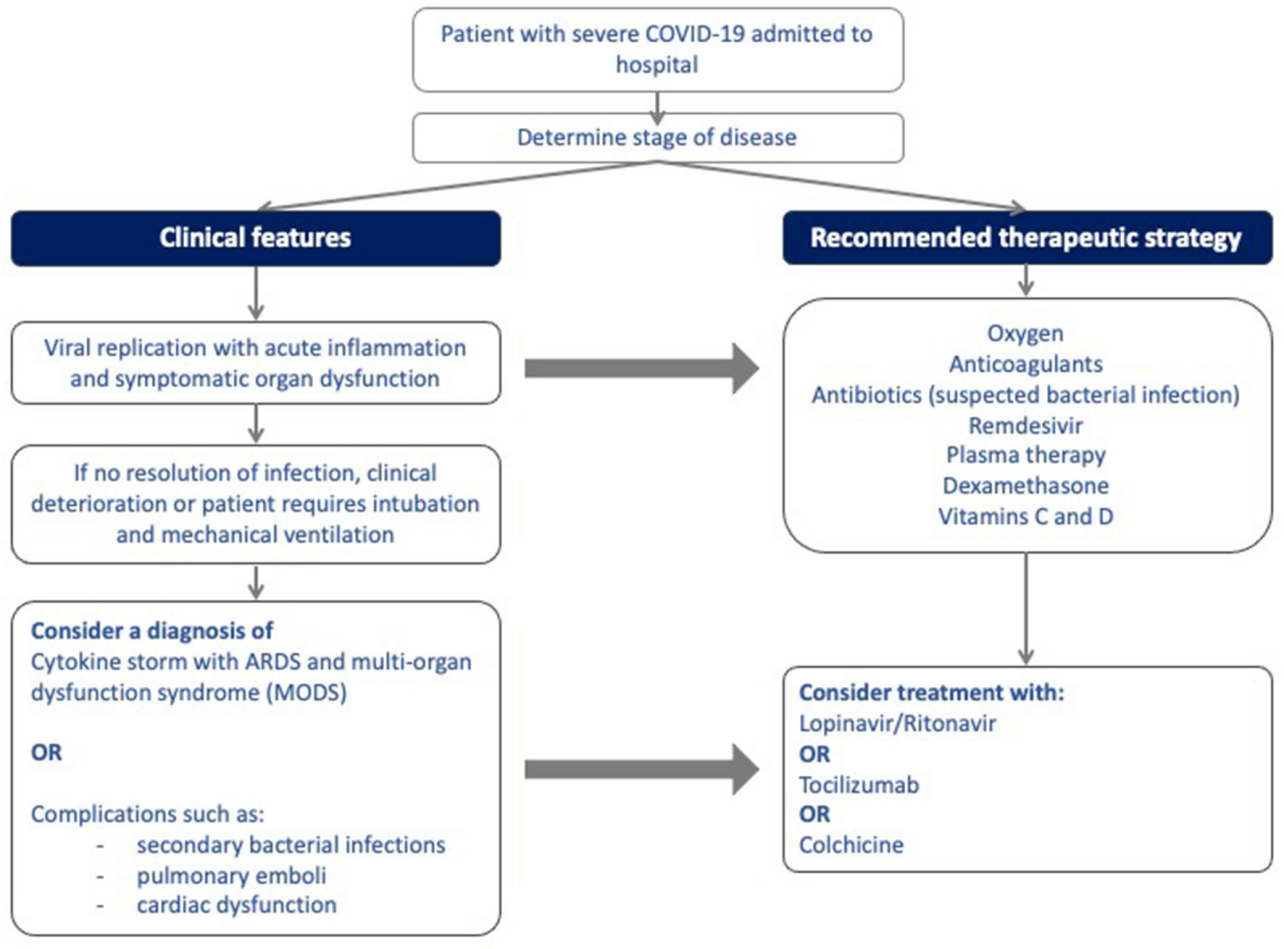
Algorithm for the stepwise initiation of treatment for patients with severe COVID-19 disease.

Figure 1 illustrates the site of action of the different treatment modalities. Figure 2 illustrates when the various modalities of therapy should be considered in patients hospitalized with COVID-19 pneumonia.

## Summary and Conclusion

The unprecedented coronavirus pandemic has challenged healthcare systems around the world with second waves causing even higher number of critically ill patients. Physicians have had to adapt rapidly to a previously unknown disorder with the introduction of novel therapeutic strategies to combat the effects of the virus and thereby reduce COVID-19 related morbidity and mortality. A rapid increase has occurred in randomized clinical trials using both established and experimental therapies, and clinicians face the daunting task of selecting treatments for patients with COVID-19 that are beneficial while avoiding potentially harmful effects. The majority of agents used have been selected to target the viral pathogen itself or the exaggerated host immune response, which may manifest as organ dysfunction or failure. In SA, the health system is currently under pressure, as is being experienced in the many parts of the world. Prudent, cost-effective decisions regarding patent management are necessary to ensure the adequate provision of effective care to all patients who require it, especially in our government funded sector where resource constraints exist.

## Author Contributions

CLH and MP completed the final preparation and editing of the manuscript. All authors contributed equally to the concept, design, and manuscript preparation.

## Conflict of Interest

The authors declare that the research was conducted in the absence of any commercial or financial relationships that could be construed as a potential conflict of interest.

## References

[1] Department of Health South Africa. COVID-19 South African Coronavirus News and Information. COVID-19 Corona Virus South African Resource Portal. (2020). Available online at: https://sacoronavirus.co.za/.

[2] John Hopkins University and Medicine. COVID-19 Map-Johns Hopkins Coronavirus Resource Center. Baltimore, MD: John Hopkins Coronavirus Resource Center (2020). p. 1. Available online at: https://coronavirus.jhu.edu/map.html

[3] Odendaal HJ Brink LT Nel DG Carstens E De Jager M Potter M. Smoking and drinking habits of women in subsequent pregnancies after specific advice about the dangers of these exposures during pregnancy. South Afr Med J. (2020) 110:1100. 10.7196/SAMJ.2020.v110i11.14667 33403986 PMC7793549

[4] Kim JS Lee JY Yang JW Lee KH Effenberger M Szpirt W. Immunopathogenesis and treatment of cytokine storm in COVID-19. Theranostics. (2021) 11:316–29. 10.7150/thno.49713 33391477 PMC7681075

[5] Byrd KM Beckwith CG Garland JM Johnson JE Aung S Cu-Uvin S. SARS-CoV-2 and HIV coinfection: clinical experience from Rhode Island, United States. J Int AIDS Soc. (2020) 23:1–7. 10.1002/jia2.25573 32657527 PMC7357287

[6] Ahn JH Choi EY. Expanded a-DROP score: a new scoring system for the prediction of mortality in hospitalized patients with community-acquired pneumonia. Sci Rep. (2018) 8:14588. 10.1038/s41598-018-32750-2 30275523 PMC6167349

[7] Liang W Liang H Ou L Chen B Chen A Li C. Development and validation of a clinical risk score to predict the occurrence of critical illness in hospitalized patients with COVID-19. JAMA Intern Med. (2020) 180:1081. 10.1001/jamainternmed.2020.2033 32396163 PMC7218676

[8] Mellet J Pepper MS. A COVID-19 vaccine: big strides come with big challenges. Vaccines. (2021) 9:1–14. 10.3390/vaccines9010039 33440895 PMC7827578

[9] Borba MGS Val FFA Sampaio VS Alexandre MAA Melo GC Brito M. Effect of high vs. low doses of chloroquine diphosphate as adjunctive therapy for patients hospitalized with severe acute respiratory syndrome coronavirus 2 (SARS-CoV-2) infection. JAMA Netw Open. (2020) 3:e208857. 10.1001/jamanetworkopen.2020.8857 32330277 PMC12124691

[10] Lim H-S Im J-S Cho J-Y Bae K-S Klein TA Yeom J-S. Pharmacokinetics of hydroxychloroquine and its clinical implications in chemoprophylaxis against malaria caused by plasmodium vivax. Antimicrob Agents Chemother. (2009) 53:1468–75. 10.1128/AAC.00339-08 19188392 PMC2663072

[11] Choudhary R Sharma AK. Potential use of hydroxychloroquine, ivermectin and azithromycin drugs in fighting COVID-19: trends, scope and relevance. New Microbes New Infect. (2020) 35:100684. 10.1016/j.nmni.2020.100684 32322397 PMC7175902

[12] Won J-H Lee H. The current status of drug repositioning and vaccine developments for the COVID-19 pandemic. Int J Mol Sci. (2020) 21:9775. 10.3390/ijms21249775 33371468 PMC7767501

[13] Wang M Cao R Zhang L Yang X Liu J Xu M. Remdesivir and chloroquine effectively inhibit the recently emerged novel coronavirus (2019-nCoV) *in vitro*. Cell Res. (2020) 30:269–71. 10.1038/s41422-020-0282-0 32020029 PMC7054408

[14] Sanders JM Monogue ML Jodlowski TZ Cutrell JB. Pharmacologic treatments for coronavirus disease 2019 (COVID-19). JAMA. (2020) 323:1824–36. 10.1001/jama.2020.6019 32282022

[15] Felsenstein S Herbert JA McNamara PS Hedrich CM. COVID-19: immunology and treatment options. Clin Immunol. (2020) 215:108448. 10.1016/j.clim.2020.108448 32353634 PMC7185015

[16] Geleris J Sun Y Platt J Zucker J Baldwin M Hripcsak G. Observational study of hydroxychloroquine in hospitalized patients with covid-19. N Engl J Med. (2020) 382:2411–8. 10.1056/NEJMoa2012410 32379955 PMC7224609

[17] Chen Z Hu J Zhang Z Jiang S Han S Yan D. Efficacy of hydroxychloroquine in patients with COVID-19: results of a randomized clinical trial. medRxiv. (2020) 10.1101:1–11. 10.1101/2020.03.22.20040758

[18] Chen J Liu D Liu L Liu P Xu Q Xia L. A pilot study of hydroxychloroquine in treatment of patients with common coronavirus disease-19 (COVID-19). J Zhejiang Univ. (2020) 49:215–9. 10.3785/j.issn.1008-9292.2020.03.03 PMC8800713 32391667

[19] Tang W Cao Z Han M Wang Z Chen J Sun W. Hydroxychloroquine in patients with COVID-19: an open-label, randomized, controlled trial. medRxiv. (2020) 10.1101:1–42. 10.1101/2020.04.10.2006055833264337

[20] Arshad S Kilgore P Chaudhry ZS Jacobsen G Wang DD Huitsing K. Treatment with hydroxychloroquine, azithromycin, and combination in patients hospitalized with COVID-19. Int J Infect Dis. (2020) 97:396–403. 10.1016/j.ijid.2020.06.099 32623082 PMC7330574

[21] Fiolet T Guihur A Rebeaud ME Mulot M Peiffer-Smadja N Mahamat-Saleh Y. Effect of hydroxychloroquine with or without azithromycin on the mortality of coronavirus disease 2019 (COVID-19) patients: a systematic review and meta-analysis. Clin Microbiolnfect. (2021) 27:19–27. 10.1016/j.cmi.2020.08.022 32860962 PMC7449662

[22] The Collaborative RECOVERY Group. Effect of hydroxychloroquine in hospitalized patients with covid-19. N Engl J Med. (2020) 383:2030–40. 10.1056/NEJMoa2022926 33031652 PMC7556338

[23] Repurposed Antiviral Drugs for Covid-19 — Interim WHO Solidarity Trial Results. N Engl J Med. (2020). 10.1056/NEJMoa2023184 PMC7727327 33264556

[24] Tu Y. The development of new antimalarial drugs: qinghaosu and dihydro-qinghaosu. Chin Med J. (1999) 112:976–7. 11721477

[25] Uzun T Toptas O. Artesunate: could be an alternative drug to chloroquine in COVID-19 treatment? Chin Med. (2020) 15:54. 10.1186/s13020-020-00336-8 32514287 PMC7254722

[26] Gendrot M Duflot I Boxberger M Delandre O Jardot P Le Bideau M. Antimalarial artemisinin-based combination therapies (ACT) and COVID-19 in Africa: *in vitro* inhibition of SARS-CoV-2 replication by mefloquine-artesunate. Int J Infect Dis. (2020) 99:437–40. 10.1016/j.ijid.2020.08.032 32805422 PMC7426697

[27] Bae J-Y Lee GE Park H Cho J Kim Y-E Lee J-Y. Pyronaridine and artesunate are potential antiviral drugs against COVID-19 and influenza. bioRxiv. (2020) 28:1–10. 10.1101/2020.07.28.225102

[28] Newman DJ Cragg GM Kingston DGI. Chapter 5 - natural products as pharmaceuticals and sources for lead structures^**^note: this chapter reflects the opinions of the authors, not necessarily those of the US Government. In: Wermuth CG Aldous D Raboisson P Rognan E editors. The Practice of Medicinal Chemistry. 4th ed. San Diego, CA: Academic Press (2015). p. 101–39. 10.1016/B978-0-12-417205-0.00005-5

[29] van Wyk B-E. A broad review of commercially important southern African medicinal plants. J Ethnopharmacol. (2008) 119:342–55. 10.1016/j.jep.2008.05.029 18577439

[30] Efferth T Romero MR Wolf DG Stamminger T Marin JJG Marschall M. The antiviral activities of artemisinin and artesunate. Clinnfect Dis. (2008) 47:804–11. 10.1086/591195 18699744

[31] Hirano T Murakami M. COVID-19: a new virus, but a familiar receptor and cytokine release syndrome. Immunity. (2020) 52:731–3. 10.1016/j.immuni.2020.04.003 32325025 PMC7175868

[32] Cheong DHJ Tan DWS Wong FWS Tran T. Anti-malarial drug, artemisinin and its derivatives for the treatment of respiratory diseases. Pharmacol Res. (2020) 158:104901. 10.1016/j.phrs.2020.104901 32405226 PMC7217791

[33] Lin Y Wu F Xie Z Song X Zhu Q Wei J. [Clinical study of artesunate in the treatment of coronavirus disease 2019]. Zhonghua Wei Zhong Bing Ji Jiu Yi Xue. (2020) 32:417–20. 32527344 10.3760/cma.j.cn121430-20200312-00412

[34] Grein J Ohmagari N Shin D Diaz G Asperges E Castagna A. Compassionate use of remdesivir for patients with severe covid-19. N Engl J Med. (2020) 382:2327–36. 10.1056/NEJMoa2007016 32275812 PMC7169476

[35] Wang Y Zhang D Du G Du R Zhao J Jin Y. Remdesivir in adults with severe COVID-19: a randomised, double-blind, placebo-controlled, multicentre trial. Lancet. (2020) 395:10236. 10.1016/S0140-6736(20)31022-9 32423584 PMC7190303

[36] Gordon CJ Tchesnokov EP Feng JY Porter DP Götte M. The antiviral compound remdesivir potently inhibits RNA-dependent RNA polymerase from Middle East respiratory syndrome coronavirus. J Biol Chem. (2020) 295:4773–9. 10.1074/jbc.AC120.013056 32094225 PMC7152756

[37] Frediansyah A Nainu F Dhama K Mudatsir M Harapan H. Remdesivir and its antiviral activity against COVID-19: a systematic review. Clin Epidemiol Glob Heal. (2021) 9:123–7. 10.1016/j.cegh.2020.07.011 32838064 PMC7410793

[38] Simonis A Theobald SJ Fätkenheuer G Rybniker J Malin JJ. A comparative analysis of remdesivir and other repurposed antivirals against SARS-CoV-2. EMBO Mol Med. (2021) 13:e13105. 10.15252/emmm.202013105 33015938 PMC7646058

[39] Beigel JH Tomashek KM Dodd LE Mehta AK Zingman BS Kalil AC. Remdesivir for the treatment of covid-19 — preliminary report. N Engl J Med. (2020) 383:993–4. 10.1056/NEJMoa2007764 32649078

[40] Goldman JD Lye DCB Hui DS Marks KM Bruno R Montejano R. Remdesivir for 5 or 10 days in patients with severe covid-19. N Engl J Med. (2020) 383:1827–37. 10.1056/NEJMoa2015301 32459919 PMC7377062

[41] Spinner CD Gottlieb RL Criner GJ Arribas López JR Cattelan AM Soriano Viladomiu A. Effect of remdesivir vs standard care on clinical status at 11 days in patients with moderate COVID-19. JAMA. (2020) 324:1048–57. 10.1001/jama.2020.16349 32821939 PMC7442954

[42] Nichols BE Jamieson L Zhang SRC Rao GA Silal S Pulliam JRC. The role of remdesivir in South Africa: preventing COVID-19 deaths through increasing ICU capacity. Clin infect Des. (2020) ciaa937. 10.1101/2020.06.10.20127084 32628744 PMC7454458

[43] Cipla. COVID-19 Drug, Remdesivir, Arrives in SA - Cipla South Africa. (2020). Available online at: https://www.cipla.co.za/cipla-news/covid-19-drug-remdesivir-arrives-in-sa/

[44] Lin HXJ Cho S Meyyur Aravamudan V Sanda HY Palraj R Molton JS. Remdesivir in coronavirus disease 2019 (COVID-19) treatment: a review of evidence. Infection. (2021) 1–10. 10.1007/s15010-020-01557-7 33389708 PMC7778417

[45] Wu P Morris A. Remdesivir for patients with COVID-19. Can Med Assoc J. (2021) 61:869–72. 10.1007/s00108-020-00836-7

[46] Charan J Kaur RJ Bhardwaj P Haque M Sharma P Misra S. Rapid review of suspected adverse drug events due to remdesivir in the WHO database; findings and implications. Expert Rev Clin Pharmacol. (2020) 1–9. 10.1080/17512433.2021.1856655 33252992 PMC7784780

[47] Chan KS Lai ST Chu CM Tsui E Tam CY Wong MML. Treatment of severe acute respiratory syndrome with lopinavir/ritonavir: a multicentre retrospective matched cohort study. Hong Kong Med J. (2003) 9:399–406. 14660806

[48] Chu CM. Role of lopinavir/ritonavir in the treatment of SARS: initial virological and clinical findings. Thorax. (2004) 59:252–6. 10.1136/thorax.2003.012658 14985565 PMC1746980

[49] Cao B Wang Y Wen D Liu W Wang J Fan G. A trial of lopinavir–ritonavir in adults hospitalized with severe covid-19. N Engl J Med. (2020) 382:1787–99. 10.1056/NEJMoa2001282 32187464 PMC7121492

[50] Corrao S Natoli G Cacopardo B. A trial of lopinavir–ritonavir in covid-19. N Engl J Med. (2020) 382:e68. 10.1056/NEJMc2008043 32369284

[51] Alhumaid S Al Mutair A Al Alawi Z Alhmeed N Zaidi ARZ Tobaiqy M. Efficacy and safety of lopinavir/ritonavir for treatment of Covid-19: a systematic review and meta-analysis. Trop Mednfect Dis. (2020) 5:180. 10.3390/tropicalmed5040180 33260553 PMC7768433

[52] Srinivas P Sacha GL Koval C. Antivirals for COVID-19. Cleve Clin J Med. (2020). 10.3949/ccjm.87a.ccc030 32409433

[53] Kieseier BC. The mechanism of action of interferon-β in relapsing multiple sclerosis. CNS Drugs. (2011) 25:491–502. 10.2165/11591110-000000000-00000 21649449

[54] Hojati Z Kay M Dehghanian F. Mechanism of action of interferon beta in treatment of multiple sclerosis. In: Minagar A editor. Multiple Sclerosis. San Diego, CA: Academic Press (2016). p. 365–92.

[55] Bosi E Bosi C Rovere Querini P Mancini N Calori G Ruggeri A. Interferon β-1a (IFNβ-1a) in COVID-19 patients (INTERCOP): study protocol for a randomized controlled trial. Trials. (2020) 21:939. 10.1186/s13063-020-04864-4 33225960 PMC7681191

[56] Sin W-X Li P Yeong JP-S Chin K-C. Activation and regulation of interferon-β in immune responses. Immunol Res. (2012) 53:25–40. 10.1007/s12026-012-8293-7 22411096

[57] Bagheri A Moezzi SMI Mosaddeghi P Nadimi Parashkouhi S Fazel Hoseini SM Badakhshan F. Interferon-inducer antivirals: potential candidates to combat COVID-19. Int Immunopharmacol. (2021) 91:107245. 10.1016/j.intimp.2020.107245 33348292 PMC7705326

[58] COVID-19 - Synairgen SG016 Clinical Trial Data Readout. (2020). Available online at: https://www.synairgen.com/covid-19/ (accessed July 20, 2020).

[59] EU Clinical Trials Register. A Randomised Double-Blind Placebo-Controlled Trial to Determine the Safety and Efficacy of Inhaled SNG001 (IFNβ-1a for Nebulisation) for the Treatment of Patients With Confirmed SARS-CoV-2 Infection (COVID-19) EudraCT number. (2020). Available online at: https://www.clinicaltrialsregister.eu/ctr-search/trial/2020-001023-14/GB (accessed July 20, 2020).

[60] Monk PD Marsden RJ Tear VJ Brookes J Batten TN Mankowski M. Safety and efficacy of inhaled nebulised interferon beta-1a (SNG001) for treatment of SARS-CoV-2 infection: a randomised, double-blind, placebo-controlled, phase 2 trial. Lancet Respir Med. (2020) 1–11. 33189161 10.1016/S2213-2600(20)30511-7 PMC7836724

[61] Mantlo E Bukreyeva N Maruyama J Paessler S Huang C. Antiviral activities of type I interferons to SARS-CoV-2 infection. Antiviral Res. (2020) 179:104811. 10.1016/j.antiviral.2020.104811 32360182 PMC7188648

[62] Hensley LE Fritz EA Jahrling PB Karp C Huggins JW Geisbert TW. Interferon-β 1a and SARS coronavirus replication. Emerg Infect Dis. (2004) 10:317–9. 10.3201/eid1002.030482 15030704 PMC3322919

[63] Baghaei P Dastan F Marjani M Moniri A Abtahian Z Ghadimi S. Combination therapy of IFNβ1 with lopinavir–ritonavir, increases oxygenation, survival and discharging of sever COVID-19 infected inpatients. Int Immunopharmacol. (2021) 92:107329. 10.1016/j.intimp.2020.107329 33412395 PMC7762801

[64] Rajendran K Narayanasamy K Rangarajan J Rathinam J Natarajan M Ramachandran A. Convalescent plasma transfusion for the treatment of COVID-19: systematic review. J Med Virol. (2020) 92:1475–83. 10.1002/jmv.25961 32356910 PMC7267113

[65] Alghamdi AN Abdel-Moneim AS. Convalescent plasma: a potential life-saving therapy for coronavirus disease 2019 (COVID-19). Front Public Heal. (2020) 8:437. 10.3389/fpubh.2020.00437 32903641 PMC7438749

[66] Rojas M Rodríguez Y Monsalve DM Acosta-Ampudia Y Camacho B Gallo JE. Convalescent plasma in Covid-19: possible mechanisms of action. Autoimmun Rev. (2020) 19:102554. 10.1016/j.autrev.2020.102554 32380316 PMC7198427

[67] Roback JD Guarner J. Convalescent plasma to treat COVID-19. JAMA. (2020) 323:1561–2. 10.1001/jama.2020.4940 32219429

[68] Smid M Epstein J Burnouf T Epstein J Kamel H Sibinga C. Points to consider in the preparation and transfusion of COVID-19 convalescent plasma in low- and middle- income countries. Vox Sang. (2020) 16:13–4. 10.4314/asan.v22i2.3 PMC7264781 32319102

[69] Chen L Xiong J Bao L Shi Y. Convalescent plasma as a potential therapy for COVID-19. Lance Infect Dis. (2020) 20:398–400. 10.1016/S1473-3099(20)30141-9 PMC7128218 32113510

[70] Shen C Wang Z Zhao F Yang Y Li J Yuan J. Treatment of 5 critically Ill patients with covid-19 with convalescent plasma. JAMA. (2020) 323:1582. 10.1001/jama.2020.4783 32219428 PMC7101507

[71] Zeng Q-L Yu Z-J Gou J-J Li G-M Ma S-H Zhang G-F. Effect of convalescent plasma therapy on viral shedding and survival in patients with coronavirus disease (2019). J Infect Dis. (2020) 222:38–43. 10.1093/infdis/jiaa228 32348485 PMC7197534

[72] Agarwal A Mukherjee A Kumar G Chatterjee P Bhatnagar T Malhotra P. Convalescent plasma in the management of moderate covid-19 in adults in India: open label phase II multicentre randomised controlled trial (PLACID Trial). BMJ. (2020) 371:m3939. 10.1136/bmj.m3939 33093056 PMC7578662

[73] Valk SJ Piechotta V Chai KL Doree C Monsef I Wood EM. Convalescent plasma or hyperimmune immunoglobulin for people with COVID-19: a rapid review. Cochrane Database Syst Rev. (2020) 10.1002:1–136. 10.1002/14651858.CD013600 32406927 PMC7271896

[74] Sarkar S Soni KD Khanna P. Convalescent plasma is a clutch at straws in COVID-19 management! a systematic review and meta-analysis. J Med Virol. (2020) 93:1111–8. 10.1002/jmv.26408 32776573 PMC7436491

[75] Bartoli A Gabrielli F Alicandro T Nascimbeni F Andreone P. COVID-19 treatment options: a difficult journey between failed attempts and experimental drugs. Intern Emerg Med. (2021) 10:1–28. 10.1007/s11739-020-02569-9 33398609 PMC7781413

[76] Han YJ Lee KH Yoon S Nam SW Ryu S Seong D. Treatment of severe acute respiratory syndrome (SARS), Middle East respiratory syndrome (MERS), and coronavirus disease 2019 (COVID-19): a systematic review of *in vitro, in vivo*, and clinical trials. Theranostics. (2021) 11:1207–31. 10.7150/thno.48342 33391531 PMC7738873

[77] Epstein J Burnouf T. Points to consider in the preparation and transfusion of COVID-19 convalescent plasma. Vox Sang. (2020) 115:485–7. 10.1111/vox.12939 32319102 PMC7264781

[78] SANBS. SANBS COVID-19 Convalescent Plasma Donor Registry. (2020). Available online at: https://sanbs.org.za/convalescent-plasma-donor/ (accessed June 24, 2020).

[79] Salazar G Zhang N Fu T-M An Z. Antibody therapies for the prevention and treatment of viral infections. NPJ Vaccines. (2017) 2:19. 10.1038/s41541-017-0019-3 29263875 PMC5627241

[80] Marovich M Mascola JR Cohen MS. Monoclonal antibodies for prevention and treatment of COVID-19. JAMA. (2020) 324:131–2. 10.1001/jama.2020.10245 32539093

[81] Hansen J Baum A Pascal KE Russo V Giordano S Wloga E. Studies in humanized mice and convalescent humans yield a SARS-CoV-2 antibody cocktail. Science. (2020) 369:1010–4. 10.1126/science.abd0827 32540901 PMC7299284

[82] Rogers TF Zhao F Huang D Beutler N Burns A He W. Isolation of potent SARS-CoV-2 neutralizing antibodies and protection from disease in a small animal model. Science. (2020) 369:956–63. 10.1126/science.abc7520 32540903 PMC7299280

[83] Guo Y Kawaguchi A Takeshita M Sekiya T Hirohama M Yamashita A. Potent mouse monoclonal antibodies that block SARS-CoV-2 infection. bioRxiv. (2020) 2020.10.01:1–38. 10.1101/2020.10.01.323220 33524396 PMC7846482

[84] Wang C Li W Drabek D Okba NMA van Haperen R Osterhaus ADME. A human monoclonal antibody blocking SARS-CoV-2 infection. Nat Commun. (2020) 11:2251. 10.1101/2020.03.11.987958 32366817 PMC7198537

[85] Sharun K Tiwari R Iqbal Yatoo M Patel SK Natesan S Dhama J. Antibody-based immunotherapeutics and use of convalescent plasma to counter COVID-19: advances and prospects. Expert Opin Biol Ther. (2020) 20:1033–46. 10.1080/14712598.2020.1796963 32744917

[86] Nicole H. Lilly Begins World's First Study of a Potential COVID-19 Antibody Treatment in Humans. Indianapolis, IN: Eli Lilly and Company (2020). Available online at: https://www.prnewswire.com/news-releases/lilly-begins-worlds-first-study-of-a-potential-covid-19-antibody-treatment-in-humans-301068303.html

[87] Yang L Liu W Yu X Wu M Reichert JM Ho M. COVID-19 antibody therapeutics tracker: a global online database of antibody therapeutics for the prevention and treatment of COVID-19. Antib Ther. (2020) 3:205–12. 10.1093/abt/tbaa020 33215063 PMC7454247

[88] Chen P Nirula A Heller B Gottlieb RL Boscia J Morris J. SARS-CoV-2 neutralizing antibody LY-CoV555 in outpatients with covid-19. N Engl J Med. (2021) 384:229–37. 10.1056/NEJMoa2029849 33113295 PMC7646625

[89] ACTIV-3/TICO LY-CoV555 Study group. A neutralizing monoclonal antibody for hospitalized patients with covid-19. N Engl J Med. (2020). 1–10. 10.1056/NEJMoa2033130 33356051 PMC7781100

[90] Weinreich DM Sivapalasingam S Norton T Ali S Gao H Bhore R. REGN-COV2, a neutralizing antibody cocktail, in outpatients with covid-19. N Engl J Med. (2021) 384:238–51. 10.1056/NEJMoa2035002 33332778 PMC7781102

[91] Baum A Ajithdoss D Copin R Zhou A Lanza K Negron N. REGN-COV2 antibodies prevent and treat SARS-CoV-2 infection in rhesus macaques and hamsters. Science. (2020) 370:1110–5. 10.1126/science.abe2402 33037066 PMC7857396

[92] Sharun K Tiwari R Dhama J Dhama K. Dexamethasone to combat cytokine storm in COVID-19: clinical trials and preliminary evidence. Int J Surg. (2020) 82:179–81. 10.1016/j.ijsu.2020.08.038 32896649 PMC7472975

[93] Russell CD Millar JE Baillie JK. Clinical evidence does not support corticosteroid treatment for 2019-nCoV lung injury. Lancet. (2020) 395:473–5. 10.1016/S0140-6736(20)30317-2 32043983 PMC7134694

[94] Shang L Zhao J Hu Y Du R Cao B. On the use of corticosteroids for 2019-nCoV pneumonia. Lancet. (2020) 395:683–4. 10.1016/S0140-6736(20)30361-5 32122468 PMC7159292

[95] Ledford H. Coronavirus breakthrough_ dexamethasone is first drug shown to save lives. Nature. (2020) 582:469. 10.1038/d41586-020-01824-5 32546811

[96] Recovery Collaborative Group Horby P Lim WS Emberson JR Mafham M Bell JL. Dexamethasone in hospitalized patients with covid-19 - preliminary report. N Engl J Med. (2020) NEJMoa2021436. 10.1056/NEJMoa2021436 32678530 PMC7383595

[97] Johnson RM Vinetz JM. Dexamethasone in the management of covid−19. BMJ. (2020) 370:m2648. 10.1136/bmj.m2648 32620554

[98] Montealegre-Gómez G Garavito E Gómez-López A Rojas-Villarraga A Parra-Medina R. Colchicine: a potential therapeutic tool against COVID-19. Experience of 5 patients. Reumatol Clín. (2020). 10.1016/j.reuma.2020.05.001 38620275 PMC7229928

[99] Burrage DR Koushesh S Sofat N. Immunomodulatory drugs in the management of SARS-CoV-2. Front Immunol. (2020) 11:1844. 10.3389/fimmu.2020.01844 32903555 PMC7438578

[100] Della-Torre E Della-Torre F Kusanovic M Scotti R Ramirez GA Dagna L. Treating COVID-19 with colchicine in community healthcare setting. Clin Immunol. (2020) 217:108490. 10.1016/j.clim.2020.108490 32492478 PMC7261351

[101] Deftereos S Giannopoulos G Vrachatis DA Siasos G Giotaki SG Cleman M. Colchicine as a potent anti-inflammatory treatment in COVID-19: can we teach an old dog new tricks? Eur Hear J Cardiovasc Pharmacother. (2020) 6:255. 10.1093/ehjcvp/pvaa033 32337546 PMC7197570

[102] Scarsi M Piantoni S Colombo E Airó P Richini D Miclini M. Association between treatment with colchicine and improved survival in a single-centre cohort of adult hospitalised patients with COVID-19 pneumonia and acute respiratory distress syndrome. Ann Rheum Dis. (2020) 79:1286–9. 10.1136/annrheumdis-2020-217712 32732245 PMC7509521

[103] ClinicalTrials.gov. The GReek Study in the Effects of Colchicine in Covid-19 cOmplications Prevention NCT04326790. (2020). Available online at: https://www.cochranelibrary.com/central/doi/10.1002/central/CN-02091174/full

[104] ClinicalTrials.gov. Colchicine Coronavirus SARS-CoV2 Trial (COLCORONA). (2020) Available online at: https://clinicaltrials.gov/ct2/show/NCT04322682

[105] ClinicalTrials.gov. The ECLA PHRI COLCOVID Trial: Effects of Colchicine on Moderate/High-Risk Hospitalized COVID-19 Patients Trial. (2020). Available online at: https://clinicaltrials.gov/show/NCT04328480.

[106] ClinicalTrials.gov. Colchicine Counteracting Inflammation in COVID-19 Pneumonia NCT04322565. (2020). https://clinicaltrials.gov/show/NCT04322565 (2020). Available online at: https://www.cochranelibrary.com/central/doi/10.1002/central/CN-02091065/full

[107] Deftereos SG Giannopoulos G Vrachatis DA Siasos GD Giotaki SG Gargalianos P. Effect of colchicine vs. standard care on cardiac and inflammatory biomarkers and clinical outcomes in patients hospitalized with coronavirus disease 2019: the grecco-19 randomized clinical trial. JAMA Netw Open. (2020) 3:e2013136. 10.1001/jamanetworkopen.2020.13136 32579195 PMC7315286

[108] Lopes MI Bonjorno LP Giannini MC Amaral NB Benatti MN Rezek UC. Beneficial effects of colchicine for moderate to severe COVID-19: an interim analysis of a randomized, double-blinded, placebo controlled clinical trial. medRxiv. (2020) 1–15. 10.1101/2020.08.06.20169573 PMC7868202 33542047

[109] Reyes AZ Hu KA Teperman J Wampler Muskardin TL Tardif J-C Shah B. Anti-inflammatory therapy for COVID-19 infection: the case for colchicine. Ann Rheum Dis. (2020) 1–8. 10.1136/annrheumdis-2020-219174 33293273 PMC8491433

[110] Cumhur Cure M Kucuk A Cure E. Colchicine may not be effective in COVID-19 infection; it may even be harmful? Clin Rheumatol. (2020) 39:2101–2. 10.1007/s10067-020-05144-x 32394215 PMC7213772

[111] Zhang C Wu Z Li J-W Zhao H Wang G-Q. Cytokine release syndrome in severe COVID-19: interleukin-6 receptor antagonist tocilizumab may be the key to reduce mortality. Int J Antimicrob Agents. (2020) 55:1–6. 10.1016/j.ijantimicag.2020.105954 32234467 PMC7118634

[112] Alzghari SK Acuña VS. Supportive treatment with tocilizumab for covid-19: a systematic review. J Clin Virol. (2020) 127:104380. 10.1016/j.jcv.2020.104380 32353761 PMC7194791

[113] Zhang W Zhao Y Zhang F Wang Q Li T Liu Z. The use of anti-inflammatory drugs in the treatment of people with severe coronavirus disease 2019 (COVID-19): The experience of clinical immunologists from China. Clin Immunol. (2020) 214:108393. 10.1016/j.clim.2020.108393 32222466 PMC7102614

[114] Luo P Liu Y Qiu L Liu X Liu D Li J. Tocilizumab treatment in COVID-19: a single center experience. J Med Virol. (2020) 92:814–8. 10.1002/jmv.25801 32253759 PMC7262125

[115] Xu X Han M Li T Sun W Wang D Fu B. Effective treatment of severe COVID-19 patients with tocilizumab. Proc Natl Acad Sci USA. (2020) 117:10970–5. 10.1073/pnas.2005615117 32350134 PMC7245089

[116] Toniati P Piva S Cattalini M Garrafa E Regola F Castelli F. Tocilizumab for the treatment of severe COVID-19 pneumonia with hyperinflammatory syndrome and acute respiratory failure: a single center study of 100 patients in Brescia, Italy. Autoimmun Rev. (2020) 19:102568. 10.1016/j.autrev.2020.102568 32376398 PMC7252115

[117] De Rossi N Scarpazza C Filippini C Cordioli C Rasia S Mancinelli CR. Early use of low dose tocilizumab in patients with COVID-19: a retrospective cohort study with a complete follow-up. EClinicalMedicine. (2020) 6:100459. 10.1016/j.eclinm.2020.100459 32838235 PMC7366117

[118] Biran N Ip A Ahn J Go RC Wang S Mathura S. Tocilizumab among patients with COVID-19 in the intensive care unit: a multicentre observational study. Lancet Rheumatol. (2020) 2:e603–12. 10.1016/S2665-9913(20)30277-0 32838323 PMC7428303

[119] Salvarani C Dolci G Massari M Merlo DF Cavuto S Savoldi L. Effect of tocilizumab vs standard care on clinical worsening in patients hospitalized with COVID-19 pneumonia. JAMA Intern Med. (2021) 181:24–31. 10.1001/jamainternmed.2020.6615 33080005 PMC7577199

[120] Salama C Han J Yau L Reiss WG Kramer B Neidhart JD. Tocilizumab in patients hospitalized with covid-19 pneumonia. N Engl J Med. (2021) 384:20–30. 10.1056/NEJMoa2030340 33332779 PMC7781101

[121] Hariyanto TI Hardyson W Kurniawan A. Efficacy and safety of tocilizumab for coronavirus disease 2019 (Covid-19) patients: a systematic review and meta-analysis. Drug Res. (2021). 10.1055/a-1336-2371 33401328

[122] Piano S Vettor R Angeli P Arcidiacono G Benfaremo D Bettini S. Tocilizumab for severe COVID-19 pneumonia. Lancet Rheumatol. (2020) 2:19–20. 10.1016/S2665-9913(20)30284-8 PMC7431163 32838327

[123] Merad M Martin JC. Pathological inflammation in patients with COVID-19: a key role for monocytes and macrophages. Nat Revmmunol. (2020) 20:355–62. 10.1038/s41577-020-0331-4 32376901 PMC7201395

[124] Dimopoulos G de Mast Q Markou N Theodorakopoulou M Komnos A Mouktaroudi M. Favorable anakinra responses in severe covid-19 patients with secondary hemophagocytic lymphohistiocytosis. Cell Host Microbe. (2020) 28:117–23.e1. 10.1016/j.chom.2020.05.007 32411313 PMC7221383

[125] Filocamo G Mangioni D Tagliabue P Aliberti S Costantino G Minoia F. Use of anakinra in severe COVID-19: a case report. Int J Infect Dis. (2020) 96:607–9. 10.1016/j.ijid.2020.05.026 32437934 PMC7211644

[126] Aouba A Baldolli A Geffray L Verdon R Bergot E Martin-Silva N. Targeting the inflammatory cascade with anakinra in moderate to severe COVID-19 pneumonia: case series. Ann Rheum Dis. (2020) 79:1381–2. 10.1136/annrheumdis-2020-217706 32376597

[127] Khan NA. Anakinra for severe forms of COVID-19. Lancet Rheumatol. (2020) 2:e586–7. 10.1016/S2665-9913(20)30273-3 PMC7413653 32838320

[128] Clark KEN Collas O Lachmann H Singh A Buckley J Bhagani S. Safety of intravenous anakinra in COVID-19 with evidence of hyperinflammation, a case series. Rheumatol Adv Pract. (2020) 4:rkaa040. 10.1093/rap/rkaa040 32964179 PMC7454860

[129] Huet T Beaussier H Voisin O Jouveshomme S Dauriat G Lazareth I. Anakinra for severe forms of COVID-19: a cohort study. Lancet Rheumatol. (2020) 2:e393–400. 10.1016/S2665-9913(20)30164-8 32835245 PMC7259909

[130] Cavalli G De Luca G Campochiaro C Della-Torre E Ripa M Canetti D. Interleukin-1 blockade with high-dose anakinra in patients with COVID-19, acute respiratory distress syndrome, and hyperinflammation: a retrospective cohort study. Lancet Rheumatol. (2020) 2:e325–31. 10.1016/S2665-9913(20)30127-2 32501454 PMC7252085

[131] Balkhair A Al-Zakwani I Al Busaidi M Al-Khirbash A Al Mubaihsi S BaTaher H. Anakinra in hospitalized patients with severe COVID-19 pneumonia requiring oxygen therapy: Results of a prospective, open-label, interventional study. Int J Infect Dis. (2021) 103:288–96. 10.1016/j.ijid.2020.11.149 33217576 PMC7670920

[132] Bozzi G Mangioni D Minoia F Aliberti S Grasselli G Barbetta L. Anakinra combined with methylprednisolone in patients with severe COVID-19 pneumonia and hyperinflammation: an observational cohort study. J Allergy Clinmmunol. (2020) 1–10. 10.1016/j.jaci.2020.11.006 33220354 PMC7674131

[133] Navarro-Millán I Sattui SE Lakhanpal A Zisa D Siegel CH Crow MK. Use of anakinra to prevent mechanical ventilation in severe COVID-19: a case series. Arthritis Rheumatol. (2020) 72:1990–7. 10.1002/art.41422 32602262 PMC7361793

[134] Vaduganathan M Vardeny O Michel T McMurray JJV Pfeffer MA Solomon SD. Renin–angiotensin–aldosterone system inhibitors in patients with covid-19. N Engl J Med. (2020) 382:1653–9. 10.1056/NEJMsr2005760 32227760 PMC7121452

[135] Albini A Di Guardo G Noonan DM Lombardo M. The SARS-CoV-2 receptor, ACE-2, is expressed on many different cell types: implications for ACE-inhibitor- and angiotensin II receptor blocker-based cardiovascular therapies. Intern Emerg Med. (2020) 15:759–66. 10.1007/s11739-020-02364-6 32430651 PMC7236433

[136] Albashir AAD. Renin-angiotensin-aldosterone system (RAAS) inhibitors and coronavirus disease 2019 (COVID-19). South Med J. (2021) 114:51–6. 10.14423/SMJ.0000000000001200 33398362 PMC7769064

[137] Mancia G Rea F Ludergnani M Apolone G Corrao G. Renin–angiotensin–aldosterone system blockers and the risk of covid-19. N Engl J Med. (2020) 382:2431–40. 10.1056/NEJMoa2006923 32356627 PMC7206933

[138] Reynolds HR Adhikari S Pulgarin C Troxel AB Iturrate E Johnson SB. Renin–angiotensin–aldosterone system inhibitors and risk of Covid-19. N Engl J Med. (2020) 382:2441–8. 10.1056/NEJMoa2008975 32356628 PMC7206932

[139] Annweiler C Cao Z Wu Y Faucon E Mouhat S Kovacic H. Counter-regulatory ‘Renin-Angiotensin’ system-based candidate drugs to treat covid-19 diseases in SARS-CoV-2-infected patients. Infect Disord -Drug Targets. (2020) 20:19–20. 10.2174/1871526520666200518073329 32418532

[140] Magrone T Magrone M Jirillo E. Focus on receptors for coronaviruses with special reference to angiotensin-converting enzyme 2 as a potential drug target - a perspective. Endocr Metab Immun Disord Drug Targets. (2020) 20:807–11. 10.2174/1871530320666200427112902 32338224

[141] Pang X Cui Y Zhu Y. Recombinant human ACE2: potential therapeutics of SARS-CoV-2 infection and its complication. Acta Pharmacol Sin. (2020) 41:1255–7. 10.1038/s41401-020-0430-6 32581256 PMC7313652

[142] Zoufaly A Poglitsch M Aberle JH Hoepler W Seitz T Traugott M. Human recombinant soluble ACE2 in severe COVID-19. Lancet Respir Med. (2020) 8:1154–8. 10.1016/S2213-2600(20)30418-5 33131609 PMC7515587

[143] Abd El-Aziz TM Al-Sabi A Stockand JD. Human recombinant soluble ACE2 (hrsACE2) shows promise for treating severe COVID-19. Signal Transduct Target Ther. (2020) 5:258. 10.1038/s41392-020-00374-6 33144565 PMC7607365

[144] Caputo ND Strayer RJ Levitan R. Early self-proning in awake, non-intubated patients in the emergency department: a single ED's experience during the COVID-19 pandemic. Acad Emerg Med. (2020) 27:375–8. 10.1111/acem.13994 32320506 PMC7264594

[145] Xu Q Wang T Qin X Jie Y Zha L Lu W. Early awake prone position combined with high-flow nasal oxygen therapy in severe COVID-19: a case series. Crit Care. (2020) 24:2–3. 10.1186/s13054-020-02991-7 32448330 PMC7246000

[146] Saba A. Observatory's starring role in pandemic. The Mail & Guardian. (2020). Available online at: https://mg.co.za/coronavirus-essentials/2020-06-26-observatorys-starring-role-in-pandemic/.

[147] Mendelson M Boloko L Boutall A L C Calligaro G Coccia C. Clinical management of COVID-19 : experiences of the COVID-19 epidemic from groote schuur hospital, Cape Town, South Africa. SAMJ S Afr Med J. (2020) 110:973–81. 10.7196/SAMJ.2020.v110i10.15157 33205723

[148] Patel M Chowdhury J Mills N Marron R Gangemi A Dorey-Stein Z. ROX index predicts intubation in patients with COVID-19 pneumonia and moderate to severe hypoxemic respiratory failure receiving high flow nasal therapy. medRxiv. (2020) 1–36. 10.1101/2020.06.30.20143867 PMC8404242 34548669

[149] Connors JM Levy JH. COVID-19 and its implications for thrombosis and anticoagulation. Blood. (2020) 135:2033–40. 10.1182/blood.2020006000 32339221 PMC7273827

[150] Kollias A Kyriakoulis KG Dimakakos E Poulakou G Stergiou GS Syrigos K. Thromboembolic risk and anticoagulant therapy in COVID-19 patients: emerging evidence and call for action. Br J Haematol. (2020) 189:846–7. 10.1111/bjh.16727 32304577 PMC7264537

[151] Bikdeli B Madhavan MV Jimenez D Chuich T Dreyfus I Driggin E. COVID-19 and thrombotic or thromboembolic disease: implications for prevention, antithrombotic therapy, and follow-up: JACC State-of-the-art review. J Am Coll Cardiol. (2020) 75:2950–73. 10.1016/j.jacc.2020.04.031 32311448 PMC7164881

[152] Wang J Hajizadeh N Moore EE McIntyre RC Moore PK Veress LA. Tissue plasminogen activator (tPA) treatment for COVID-19 associated acute respiratory distress syndrome (ARDS): a case series. J Thromb Haemost. (2020) 18:1752–5. 10.1111/jth.14828 32267998 PMC7262152

[153] Rico-Mesa JS Rosas D Ahmadian-Tehrani A White A Anderson AS Chilton R. The role of anticoagulation in COVID-19-induced hypercoagulability. Curr Cardiol Rep. (2020) 22:53. 10.1007/s11886-020-01328-8 32556892 PMC7298694

[154] Tang N Bai H Chen X Gong J Li D Sun Z. Anticoagulant treatment is associated with decreased mortality in severe coronavirus disease 2019 patients with coagulopathy. J Thromb Haemost. (2020) 18:1094–9. 10.1111/jth.14817 32220112 PMC9906401

[155] Shen L Qiu L Liu D Wang L Huang H Ge H. The association of low molecular weight heparin use and in-hospital mortality among patients hospitalized with COVID-19. Cardiovasc Drugs Ther. (2021) 1–8. 10.1007/s10557-020-07133-3 33394360 PMC7779648

[156] Martinelli I Ciavarella A Abbattista M Aliberti S De Zan V Folli C. Increasing dosages of low-molecular-weight heparin in hospitalized patients with Covid-19. Intern Emerg Med. (2021) 1–7. 10.1007/s11739-020-02585-9 33389568 PMC7778858

[157] Khoury M Cuenca J Cruz FF Figueroa FE Rocco PRM Weiss DJ. Current status of cell-based therapies for respiratory virus infections: applicability to COVID-19. Eur Respir J. (2020) 55:2000858. 10.1183/13993003.00858-2020 32265310 PMC7144273

[158] Barros I Silva A de Almeida LP Miranda CO. Mesenchymal stromal cells to fight SARS-CoV-2: taking advantage of a pleiotropic therapy. Cytokine Growth Factor Rev. (2020) 1–20. 10.1016/j.cytogfr.2020.12.002 33397585 PMC7836230

[159] Leng Z Zhu R Hou W Feng Y Yang Y Han Q. Transplantation of ACE2(-) mesenchymal stem cells improves the outcome of patients with COVID-19 pneumonia. Aging Dis. (2020) 11:216–28. 10.14336/AD.2020.0228 32257537 PMC7069465

[160] Meng F Xu R Wang S Xu Z Zhang C Li Y. Human umbilical cord-derived mesenchymal stem cell therapy in patients with COVID-19: a phase 1 clinical trial. Signal Transduct Target Ther. (2020) 5:172. 10.1038/s41392-020-00286-5 32855385 PMC7450163

[161] Shu L Niu C Li R Huang T Wang Y Huang M. Treatment of severe COVID-19 with human umbilical cord mesenchymal stem cells. Stem Cell Res Ther. (2020) 11:361. 10.1186/s13287-020-01875-5 32811531 PMC7432540

[162] Zhao Q Ren H Han Z. Mesenchymal stem cells: Immunomodulatory capability and clinical potential in immune diseases. J Cellmmunother. (2016) 2:3–20. 10.1016/j.jocit.2014.12.001

[163] Weiss ARR Dahlke MH. Immunomodulation by Mesenchymal Stem Cells (MSCs): mechanisms of action of living, apoptotic, and dead MSCs. Front Immunol. (2019) 10:1191. 10.3389/fimmu.2019.01191 31214172 PMC6557979

[164] Sadeghi S Soudi S Shafiee A Hashemi SM. Mesenchymal stem cell therapies for COVID-19: Current status and mechanism of action. Life Sci. (2020) 262:118493. 10.1016/j.lfs.2020.118493 32979360 PMC7510562

[165] Singh S Chakravarty T Chen P Akhmerov A Falk J Friedman O. Allogeneic cardiosphere-derived cells (CAP-1002) in critically ill COVID-19 patients: compassionate-use case series. Basic Res Cardiol. (2020) 115:36. 10.1007/s00395-020-0795-1 32399655 PMC7214858

[166] Basiri A Pazhouhnia Z Beheshtizadeh N Hoseinpour M Saghazadeh A Rezaei N. Regenerative medicine in COVID-19 treatment: real opportunities and range of promises. Stem Cell Rev Rep. (2020) 1–13. 10.1007/s12015-020-09994-5 32564256 PMC7305935

[167] Keller MD Harris KM Jensen-Wachspress MA Kankate VV Lang H Lazarski CA. SARS-CoV-2–specific T cells are rapidly expanded for therapeutic use and target conserved regions of the membrane protein. Blood. (2020) 136:2905–17. 10.1182/blood.2020008488 33331927 PMC7746091

[168] Lu J Wu J Tian J Wang S. Role of T cell-derived exosomes in immunoregulation. Immunol Res. (2018) 66:313–22. 10.1007/s12026-018-9000-0 29804198

[169] Anel A Gallego-Lleyda A de Miguel D Naval J Martínez-Lostao L. Role of exosomes in the regulation of T-cell mediated immune responses and in autoimmune disease. Cells. (2019) 8:154. 10.3390/cells8020154 PMC6406439 30759880

[170] Market M Angka L Martel AB Bastin D Olanubi O Tennakoon G. Flattening the COVID-19 curve with natural killer cell based immunotherapies. Front Immunol. (2020) 11:1512. 10.3389/fimmu.2020.01512 32655581 PMC7324763

[171] Zheng M Gao Y Wang G Song G Liu S Sun D. Functional exhaustion of antiviral lymphocytes in COVID-19 patients. Cell Mol Immunol. (2020) 17:533–5. 10.1038/s41423-020-0402-2 32203188 PMC7091858

[172] Diao B Wang C Tan Y Chen X Liu Y Ning L. Reduction and functional exhaustion of t cells in patients with coronavirus disease 2019 (COVID-19). Front Immunol. (2020) 11:827. 10.3389/fimmu.2020.00827 32425950 PMC7205903

